# Atrial fibrillation hospitalization is associated with exposure to fine particulate air pollutants

**DOI:** 10.1186/s12940-019-0554-7

**Published:** 2019-12-30

**Authors:** Hsiu Hao Lee, Shih Chun Pan, Bing Yu Chen, Shih Hsiang Lo, Yue Leon Guo

**Affiliations:** 1 Department of Internal Medicine, Taipei City Hospital, Zhongxing Branch, No. 145, Zhengzhou Rd., Datong Dist, Taipei City, 10341 Taiwan; 2 Institution of Occupational Medicine and Industrial Hygiene, National Taiwan University College of Public Health, Room 703, No. 17, Xu-Zhou Road, Taipei, 100 Taiwan; 3 National Institute of Environmental Health Sciences, National Health Research Institutes, 10 F, Bldg F, 3 Yuanqu Street, Taipei, 11503 Taiwan

**Keywords:** Air pollution, Atrial fibrillation, Particulate matter, Case–crossover

## Abstract

**Background:**

Although air pollutants have been associated with cardiopulmonary mortality, their effects on the occurrence of atrial fibrillation (Afib) remain unclear. This study examined the association between ambient air pollutants and Afib occurrence.

**Methods:**

Using a representative sample from the National Health Insurance Database of Taiwan, we applied a case–crossover study design to explore the associations between air pollutants and patients hospitalized with Afib from 2006 to 2011. The event day was when a patient was hospitalized with Afib, and the control days were the same days of the following weeks of the same month. The association between Afib occurrence and levels of ambient air pollutants (including particulate matter [PM] 2.5 PM_10_, NO_2_, SO_2_, and O_3_) was examined after adjusting for temperature and relative humidity. A two-pollutant model was used to examine the effect of the second pollutant when the first pollutant was determined to be significantly related to Afib.

**Results:**

During 2006–2011, 670 patients hospitalized with the first onset of Afib were identified. The occurrence of Afib was associated with PM_2.5_, in which a 22% (95% confidence interval = 3–44%) increase was related to an interquartile range increase (26.2 μg/m3) on the same day and a 19% (95% confidence interval = 0–40%) increase on the second day. A two-pollutant model was applied, and the results indicated that the effect of PM_2.5_ was significantly associated with the occurrence of Afib. Patients aged over 65 years with DM and with hyperlipidemia were more susceptible to the effect of PM_2.5_.

**Conclusions:**

In conclusion, the occurrence of Afib was associated with short-term exposure to fine particulate air pollutants in the general population.

## Introduction

Atrial fibrillation (Afib) is the most commonly sustained cardiac arrhythmia, and it occurs in approximately 2% of the general population [1, 2]. Afib is associated with reduced quality of life, increased thromboembolic events, and increased death rates [3–5]. In particular, Afib-induced stroke is often severe and results in long-term disability or death [1]. Although advancements in the diagnosis and treatment of Afib have improved its prognosis, understanding the causes of Afib can help understand the methods of preventing this severe medical condition. In recent years, fine particulate matter < 2.5 mm in aerodynamic diameter (PM_2.5_) has been increasingly associated with the onset or attack of cardiac events including sudden cardiac death, heart failure, and myocardial infarctions [6–8]. PM_2.5_ is produced through direct emissions from local and regional sources such as motor vehicles in addition to upwind secondary particles from burning fossil fuels [9]. A meta-analysis showed that a 10-μg/m^3^ increment in PM_2.5_ was associated with a 1.04% (95% confidence interval [CI] = 0.52–1.56%) increase in the risk of all-cause mortality and 0.84% (95% CI = 0.41–1.28%) increase in the risk of cardiovascular mortality [10]. In addition, stroke was associated with PM_2.5_, with increased risks of 1.1% (95% CI = 1.1–1.2%) per 10 μg/m^3^ increase in PM_2.5_ [11]. However, whether air pollution induces Afib in the general population is uncertain. In the current study, we determined whether exposure to ambient air pollutants is associated with an increased risk of Afib hospitalization. We used a representative national health database to examine this hypothesis.

## Methods

Our data source was the National Health Insurance (NHI) program in Taiwan. The NHI program, which was implemented on March 1, 1995, is a compulsory health insurance program. Under this nationwide program, up to 99% of the nation’s population receive myriad health care services, including outpatient services, inpatient care, traditional Chinese medicine, dental care, prenatal care or obstetric services, physical therapy, preventive health care, home care, and rehabilitation. The NHI maintains a comprehensive, validated patient database containing information on patient diagnoses and drug prescriptions. The quality of its information on prescription use, diagnoses, and hospitalizations is excellent [12] The NHI sample files, which are constructed and managed by the National Health Research Institutes, consist of comprehensive use and enrollment information for a randomly selected sample of 1 million NHI beneficiaries, representing approximately 5% of enrollees in Taiwan in 2000. A multistage stratified systematic sampling design was used to create the sample, and no statistically significant differences in sex or age were observed between the sample group and all enrollees. All information allowing a specific patient to be identified is encrypted. The confidentiality of the data is maintained in accordance with the data protection regulations of the Bureau of National Health Insurance (BNHI).

Patients diagnosed with Afib (based on the International Classification of Diseases, Ninth Revision, clinical modification code 437.31) for the first time from the inpatient claims database between January 1, 2006 and December 31, 2011 were retrieved as potential study participants. Patients with any previous inpatient and outpatient diagnosis of Afib before this admission were excluded. The Institutional Review Board of the Taiwan National Health Research Institutes approved this study (IRB No.: NHRI-107-EMSP02). Ultimately, 670 patients with Afib hospitalization for the first time were analyzed.

Complete air-quality-related data from the Taiwanese Environmental Protection Administration were retrieved from 77 fixed-site air quality monitoring stations from 2006 to 2011. Each station routinely monitored hourly criteria air pollutants, including CO (parts per million, ppm), NO (parts per billion, ppb), NO2 (ppb), NOx (ppb), SO2 (ppb.), O3 (ppb), O3 8 h maximum (ppb; defined as the maximum average level of ozone for 8 consecutive h/d), PM2.5 (μg/m3), and PM10 PM2.5 (μg/m3), as well as ambient temperature (°C). The monitoring stations were fully automated, and they routinely monitored the levels of pollutants including SO2 (through ultraviolet fluorescence), PM (through beta-ray absorption), NO2 (through ultraviolet fluorescence), CO (through nondispersive infrared photometry), and O3 (through ultraviolet photometry). PM2.5 concentrations in Taiwan have been measured continuously since 2006. The availability of the monitoring network for PM2.5 provided an opportunity to investigate the effect of PM2.5 on the onset of Afib. For each day, hourly air pollution data were obtained from the monitoring stations. The 24-h average level of each pollutant was computed according to the hourly mean levels of the day. For each day, for any individual pollutant with 8 or more missing hourly average values, the daily average level was treated as a missing value.

Data were analyzed using the case–crossover technique, which is an alternative to using Poisson time-series regression models for studying the short-term effects of air pollutants [13]. A time-stratified approach was used for case–crossover analysis. Time was stratified into separate months so that referent days could be selected as the days falling on the same day of the week within the same month to serve as the index day. Air pollution levels during the case period were compared with exposures occurring on all referent days. This stratified referent selection scheme minimizes bias because of stagnant air pollution time-series data [14]. The associations between Afib and air pollutants were estimated through conditional logistic regression. All statistical analyses were performed using the SAS package (version 9.3, SAS Institute Inc., Cary, NC, USA). Both single- and two-pollutant models were fitted with various combinations of pollutants (up to two pollutants per model) to assess the stability of the effect of air pollutants. Levels of exposure to air pollutants were entered into the models as continuous variables. The daily average temperature, as a meteorological variable that might play a confounding role, was included in the model. Odds ratios (ORs) and their 95% confidence intervals (Cis) were calculated for the interquartile range (IQR) differences (between the 25th and the 75th percentile). The temporal association between air pollutants and hospital admission for Afib development was further stratified according to time lags. Summary estimates for lag zero (Day 1) refer to the risk of an event per increment in air pollution on the day of the event. Lag 1 (Day 2) refers to risk estimates per increment in air pollutant concentrations 1 day before the event. Single lags from 0 to 4 (Day 1, 2, 3, 4 and 5) and cumulative lag 0–4 (Day1–5) were analyzed. Potential risk factors for the development of Afib [2, 15, 16], including age, diabetes mellitus, hypertension, dyslipidemia, COPD, congestive heart failure, coronary artery disease, and chronic kidney disease, were incorporated.

## Result

The characteristics and comorbidities of patients included in this study are presented in Table 1. During the 6 years of the study, 670 patients (51.2% men) were diagnosed with Afib for the first time and were hospitalized. Temperature, relative humidity, and monitored criteria air pollutants during 2006–2011 are provided in Table 2. Spearman correlation coefficients among these variables are presented in Table 3. There was a certain degree of correlation among PM2.5 and other pollutants, especially PM2.5 and PM10 (*r* = 0.95), PM2.5 and CO (*r* = 0.66), PM2.5 and NO2 (*r* = 0.61), PM2.5 and NOx (*r* = 0.52), PM2.5 and O3 8 h maximum (*r* = 0.62), and PM2.5 and SO2 (*r* = 0.63).

**Table 1:** The characteristics and comorbidities of study population (*n* = 670)

Variables	N	(%)	Mean ± SD
Age	670	(100.0)	70.5 ± 14.0
< 65	188	(28.1)	
≥ 65	482	(71.9)	
Gender			
Male	343	(51.2)	
Comorbidity			
Coronary artery disease	447	(66.7)	
Congestive heart failure	287	(42.8)	
Hypertension	529	(79.0)	
Hyperlipidemia	257	(38.4)	
COPD	112	(16.7)	
Diabetes mellitus	213	(31.8)	
Chronic kidney disease	57	(8.5)

**Table 2:** Distribution of temperature, relative humidity, and air pollutants during 2006–2011

Pollutant	Min	Mean	SD	Max	Percentiles			IQR
					25th	50th	75th	
PM_2.5_, μg/m^3^	1.53	34.04	18.79	164.21	19.13	30.14	45.37	26.23
PM_10_, μg/m^3^	5.19	60.08	33.63	1214.07	35.71	52.28	78.21	42.49
CO, ppm	0.04	0.44	0.16	2.42	0.33	0.43	0.54	0.21
NO, ppb	0.65	4.10	2.87	77.01	2.49	3.28	4.69	2.20
NO_2_, ppb	0.61	15.24	6.32	56.48	10.51	14.57	19.05	8.54
NO_x_, ppb	2.30	19.4	8.19	129.92	13.69	17.79	23.25	9.56
SO_2_, ppb	0.26	3.87	1.71	25.14	2.72	3.51	4.58	1.86
O_3_, ppb	0.73	29.17	10.53	86.29	21.35	27.81	35.70	14.35
O_3,_ max 8 h, ppb	1.41	47.81	18.35	140.25	33.78	45.30	59.97	26.19
AMB_TEMP, °C	2.31	24.01	4.81	52.15	20.42	25.05	28.11	7.69
RH, %	1.49	74.91	7.09	98.40	70.66	74.97	79.32	8.66

*Min* Minimum, *Max* Maximum, *SD* Standard deviation, *IQR* Interquartile range

**Table 3:** Spearman correlation coefficients of temperature, relative humidity, and air pollutants

	PM_2.5_	PM_10_	CO	NO	NO_2_	NOx	O_3_	O_3_^a^	SO_2_	RH	TEMP
PM_2.5_	1	0.95	0.66	0.07	0.61	0.52	0.47	0.62	0.63	−0.33	−0.31
PM_10_		1	0.61	0.07	0.59	0.50	0.47	0.61	0.64	−0.38	−0.32
CO			1	0.47	0.82	0.84	0.16	0.28	0.47	−0.07	− 0.60
NO				1	0.52	0.70	− 0.52	− 0.27	0.28	0.08	−0.24
NO_2_					1	0.95	0.06	0.23	0.54	−0.12	−0.56
NOx						1	−0.06	0.13	0.51	−0.08	−0.56
O_3_							1	0.88	0.23	−0.32	−0.04
O_3,_ max 8 h								1	0.41	−0.39	0.05
SO_2_									1	−0.33	−0.04
RH										1	−0.03
TEMP											1

^a^8 h maximum

*RH* Relative humidity, *TEMP* Ambient temperature

The adjusted OR for the occurrence of Afib in a single-pollutant model is shown in Table 4. The occurrence of Afib was associated with PM2.5, in which a 22% (95% CI = 3–44%) increase was related to an IQR increase in PM2.5 (26.2 μg/m3) on the same day (lag 0) and a 19% (95% CI = 0–40%) increase on the second day. None of the other pollutants was associated with Afib occurrence. A two-pollutant model was applied, and the results indicated that the effect of PM2.5 remained significantly associated with Afib occurrence, with the addition of any of the climate factors or air pollutants (Table 5). PM10 was not used in the two-pollutant analysis because of its high correlation (*r* = 0.95) with PM2.5.

**Table 4:** Adjusted OR* for atrial fibrillation onset in a single-pollutant model^a^ in the case-crossover study in Taiwan during 2006–2011

Day	Pollutants		PM_2.5–10_		PM_10_		CO		NO	
	PM_2.5_									
	OR	95% CI	OR	95% CI	OR	95% CI	OR	95% CI	OR	95% CI
1	1.22	(1.03–1.44)	1.00	(0.91–1.09)	1.08	(0.95–1.24)	0.94	(0.84–1.06)	0.98	(0.93–1.04)
2	1.19	(1.00–1.40)	1.00	(0.92–1.10)	1.08	(0.94–1.25)	1.02	(0.91–1.14)	1.03	(0.98–1.09)
3	1.02	(0.86–1.21)	0.92	(0.80–1.06)	0.95	(0.80–1.14)	1.00	(0.90–1.12)	1.02	(0.97–1.08)
4	0.96	(0.81–1.14)	1.03	(0.95–1.11)	1.02	(0.88–1.17)	0.99	(0.88–1.11)	1.02	(0.97–1.07)
5	1.00	(0.85–1.18)	1.00	(0.90–1.10)	1.00	(0.86–1.16)	0.97	(0.86–1.09)	0.98	(0.93–1.04)
1–5	1.29	(0.99–1.68)	0.99	(0.83–1.18)	1.15	(0.89–1.48)	1.20	(0.98–1.46)	1.04	(0.96–1.14)
	NO_2_		NOx		SO_2_		O_3_		O_3__8h_max	
	OR	95%CI	OR	95%CI	OR	95%CI	OR	95%CI	OR	95%CI
1	0.95	(0.81–1.11)	0.94	(0.84–1.06)	1.01	(0.90–1.14)	1.02	(0.87–1.19)	0.98	(0.83–1.16)
2	0.99	(0.84–1.16)	1.02	(0.91–1.14)	1.01	(0.90–1.14)	1.09	(0.94–1.27)	1.10	(0.93–1.29)
3	0.92	(0.79–1.08)	1.00	(0.90–1.12)	0.92	(0.81–1.04)	1.07	(0.92–1.24)	1.02	(0.87–1.20)
4	0.97	(0.83–1.14)	0.99	(0.88–1.11)	1.00	(0.89–1.13)	1.02	(0.88–1.18)	1.02	(0.87–1.20)
5	0.95	(0.81–1.12)	0.97	(0.86–1.09)	0.98	(0.87–1.11)	1.02	(0.88–1.18)	0.94	(0.80–1.10)
1–5	0.93	(0.72–1.20)	1.00	(0.83–1.21)	1.02	(0.83–1.26)	1.16	(0.92–1.46)	1.08	(0.83–1.41)

OR calculated for interquartile range increases of PM2.5 (26.2 μg/m^3^), PM10 (42.5 μg/m^3^), CO (0.2 ppm), NO (2.2 ppb), NO_2_ (8.5 ppb), NOx (9.6 ppb), SO_2_ (1.9 ppb), O_3_ (14.4 ppb), O_3,_ max 8 h (26.2 ppb), and ambient temperature (AMB_TEMP) (7.7 °C)

^a^All models adjusted for temperature and relative humidity

**Table 5:** Adjusted OR^a^ for atrial fibrillation onset in two-pollutant model^b^ using case-crossover study in Taiwan during 2006–2011

Pollutant	Day	OR^a^	(95% CI)
PM_2.5_ adj. CO	1	1.43	(1.14–1.79)
	2	1.18	(0.94–1.47)
	3	0.94	(0.75–1.18)
	4	0.87	(0.70–1.09)
	5	1.00	(0.83–1.20)
	1–5	1.39	(1.03–1.86)
PM_2.5_ adj. NO	1	1.19	(1.01–1.41)
	2	1.13	(0.95–1.34)
	3	1.00	(0.84–1.19)
	4	0.95	(0.81–1.13)
	5	1.00	(0.85–1.18)
	1–5	1.27	(0.97–1.66)
PM_2.5_ adj. NO_2_	1	1.33	(1.10–1.61)
	2	1.25	(1.03–1.52)
	3	1.09	(0.89–1.33)
	4	0.98	(0.81–1.18)
	5	1.03	(0.85–1.25)
	1–5	1.57	(1.14–2.18)
PM_2.5_ adj. SO_2_	1	1.25	(1.03–1.52)
	2	1.21	(0.99–1.47)
	3	1.11	(0.91–1.36)
	4	0.96	(0.79–1.16)
	5	1.01	(0.83–1.23)
	1–5	1.42	(1.03–1.96)
PM_2.5_ adj. O_3_	1	1.22	(1.02–1.45)
	2	1.14	(0.95–1.36)
	3	0.98	(0.81–1.78)
	4	0.96	(0.80–1.15)
	5	0.98	(0.82–1.18)
	1–5	1.25	(0.93–1.68)
PM_2.5_ adj. O_3,_ max 8 hr	1	1.30	(1.07–1.57)
	2	1.15	(0.95–1.40)
	3	1.00	(0.82–1.22)
	4	0.95	(0.79–1.56)
	5	1.04	(0.86–1.27)
	1–5	1.37	(0.99–1.91)

^a^OR calculated for an interquartile range increases of PM_2.5_ (26.2 μg/m^3^), CO (0.2 ppm), SO_2_ (1.9 ppb), NO (2.2 ppb), NO_2_ (8.5 ppb), NOx (9.6 ppb), O_3_ (14.3 ppb), O_3_* (26.2 ppb) and PM_10_ (42.5 μg/m^3^)

^b^All models adjusted for temperature and relative humidity

**Fig. 1:**
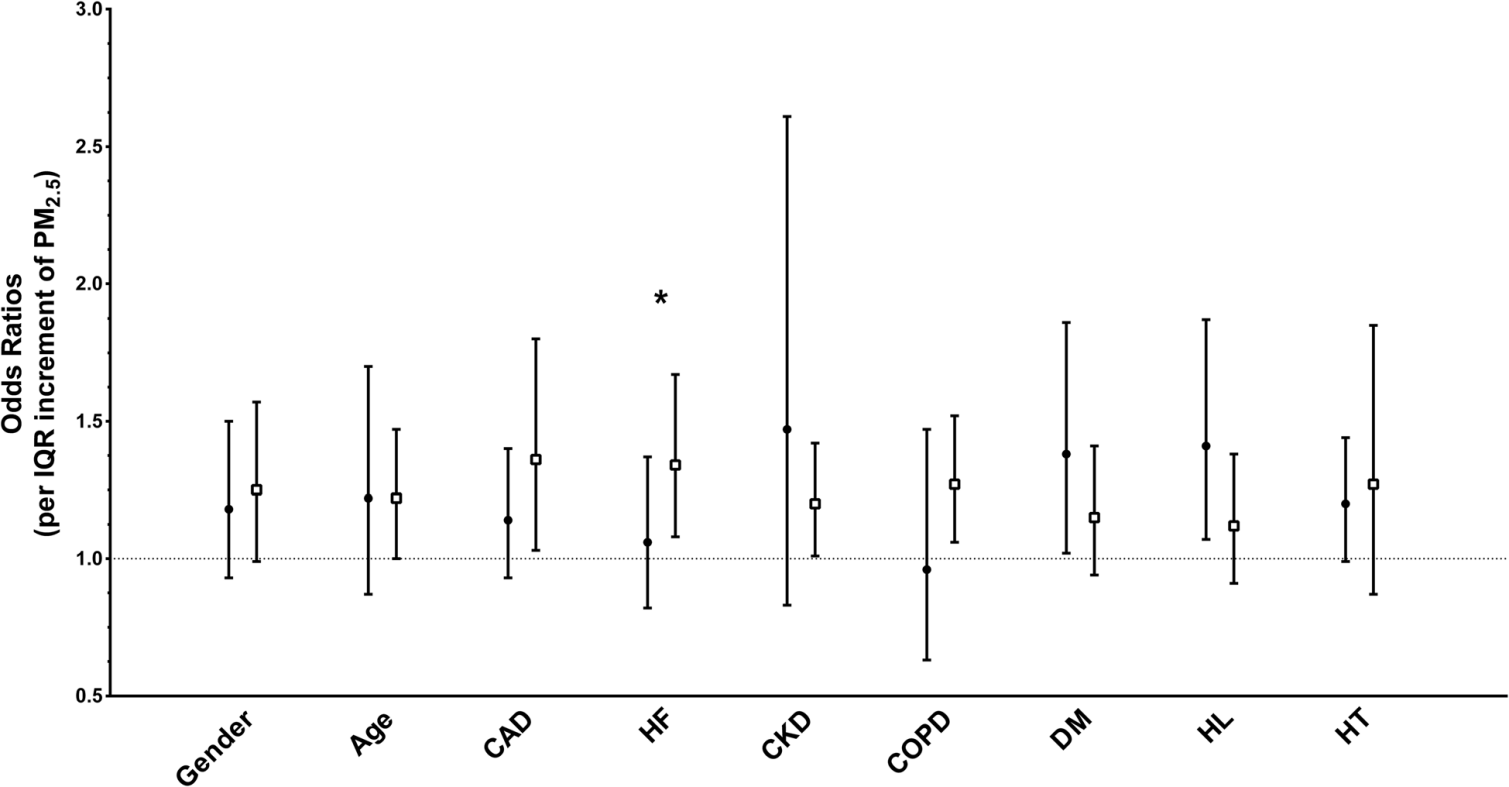
Adjusted OR for atrial fibrillation onset in subgroup analysis using case-crossover study in Taiwan during 2006–2011. CAD: coronary artery disease; HF: heart failure; CKD: chronic obstructive pulmonary disease; DM: diabetes mellitus; HL: hyperlipidemia; HT: hypertension. ˙: female; age < 65; with comorbidity. □: male; age ≥ 65; without comorbidity. *: interaction *P* value less than 0.05

The multivariate stratified analysis for Afib development is shown in Fig. 1. Patients aged greater than 65 years, without coronary artery disease, without chronic kidney disease, without COPD, with DM, and with hyperlipidemia, might be susceptible to the effect of PM2.5. Patients without congestive heart failure were more susceptible to the effect of PM2.5 than those with congestive heart failure.

## Discussion

This is the first study to demonstrate that PM_2.5_ might trigger Afib in the general population with no previously diagnosed Afib. The average levels of PM_2.5_ and IQR during the study period were 34 and 26.2 μg/m^3^, respectively. The identified association between PM_2.5_ and Afib occurrence cannot be explained by climatic factors or the other air pollutants.

This is not the first study to examine the potential association between exposure to PM and Afib development. Bunch determined that Afib hospitalization did not increase with short-term elevations in exposure to PM2.5 (mean 10–11 μg/m3). Most studies have investigated patients wearing implantable cardioverter defibrillators (ICDs) and discovered associations between particulate air pollutants and attacks of Afib or ventricular fibrillation [17–22]. A study on ICD patients revealed that the possibility of Afib increased by 26% (95% CI 8–47%) for each 6.0-μg/m3 increase in PM2.5 [23]. However, those wearing ICDs were distinct from the general population. First, heart failure is one of the most common causes of ICD implantation for the primary or secondary prevention of ventricular arrhythmias. A high risk of Afib has been reported among patients with heart failure [24, 25]. Most of the aforementioned studies have included patients with coronary artery disease. Ischemic heart disease events increase with PM exposure [26]; thus, they may affect Afib incidence, potentially biasing an analysis of atrial arrhythmias. Therefore, the findings indicated that the effects of PM on the occurrence of Afib in patients wearing ICDs observed in highly susceptible groups cannot be easily extrapolated to the general population.

Potential intriguing mechanisms may increase Afib risk with particulate air pollution exposure. Acute alterations in autonomic tone and impaired heart rate variability have been documented in humans [27–29] and animals [30, 31] exposed to PM2.5. Moreover, particulate air pollution is linked to C-reactive protein, a marker of inflammation [32–34]. Changes in autonomic tone [35, 36], inflammation and oxidative stress [37, 38], atrial ischemia [39], and atrial pressure [39, 40] may induce Afib. Other admissions because of cardiac ischemia were increased by PM2.5 [41, 42]. An increase in PM in patients with moderate or severe heart failure increases right ventricular pressure, which in turn increases right atrial pressure [43]. Therefore, particulate air pollution causes Afib.

As already stated, the causes of Afib onset, whether initial or recurrent, were complex and numerous. Individuals with chronic comorbid health conditions may have an increased risk of cardiovascular morbidity and mortality associated with air pollutants levels [44]. The subgroup analysis study revealed that patients aged over 65 years without coronary artery disease, chronic kidney disease, and COPD but with DM and hyperlipidemia might be susceptible to the adverse effects of PM2.5. Patients without congestive heart failure were more susceptible to the adverse effect of PM2.5 than those with congestive heart failure. As mentioned in related reports [44, 45], the frailty of older adults, among whom the prevalence of chronic cardiopulmonary diseases is higher, is the most likely reason why patients aged over 60 years have a higher risk of Afib admission due to the effects of PM2.5. A relationship between DM and Afib [46] exists, and this paper suggests that patients with DM, reduced heart rate variability, increased C-reactive protein levels, and elevated inflammatory markers [47] be aware of the effects of PM2.5 to prevent Afib admission. Inconsistent results may derive from factors related to the disease itself. Patients diagnosed with hyperlipidemia are more likely to use statins; statin use may reduce the risk of Afib [44]. Causes of Afib onset other than PM2.5 might have a less substantial effect on patients with hyperlipidemia. Therefore, the effects of PM2.5 on Afib onset became significant for those with hyperlipidemia. Patients with cardiovascular disease, CKD, and COPD are more likely to reduce their exposure to air pollution and take heart rhythm control medications, thus reducing their risk of developing arrhythmia. Patients diagnosed with congestive heart failure are more likely to use beta-blockers, which may reduce the risk of Afib [48]. Although the results of the subgroup analysis in our study were inconclusive, our paper argues for randomized control trials to ascertain which subgroup is more susceptible to the effect of PM2.5 on AF development.

This study has several strengths. First, Afib cases and the study population were retrieved from the BNHI database, which covers most of the Taiwanese population. By June 2014, 23 million people in Taiwan were enrolled in the NHI program, yielding a coverage rate of > 99.5%. The quality of information from the NHI database on prescription use, diagnoses, and hospitalizations is excellent [49]. To ensure the accuracy of the claims files, the BNHI conducts quarterly expert reviews on a random sample of every 50–100 ambulatory and inpatient claims. False reports of diagnostic information result in a hefty penalty from the BNHI [12]. Second, we did not include patients with a previous diagnosis of Afib; therefore, the observation of increased Afib in the present study was likely the first onset of Afib. Third, only those diagnosed with Afib in emergency departments who were then hospitalized were counted among our cases. Therefore, the diagnosis was highly valid.

This study has some potential limitations. First, several possible variables associated with Afib development were not considered, including blood pressure, smoking, family history, and alcohol consumption. Communicating with patients directly was impractical for us because the data were anonymized. However, because each participant served as a lone control in this case–crossover study, numerous variables were unlikely to bias our overall findings. Such factors could predispose the onset of Afib, but this is unlikely; nevertheless, because control days were selected from the same day of various weeks of the month, probable random errors, not systematic errors, could be introduced. Thus, the observed association might have been inclined toward the null hypothesis. Second, because the NHI in Taiwan was established on March 1, 1995, participants in this study with an Afib diagnosis before that date could not be identified. However, patients who were diagnosed with Afib before 1995 and did not seek medical attention between March 1, 1995 and December 31, 2005 may be rare.

## Conclusions

In conclusion, compared with that at 19 μg/m3, ambient PM2.5 at 45 μg/m3 was associated with an approximately 22% increase in Afib in the general population. This equates to an 8.6% increase in Afib per 10 μg/m3 increase in PM2.5. Preventive strategies are warranted to reduce the risk of Afib when PM2.5 is elevated.

## Data Availability

Weather and air pollutants data are available from Environmental Protection Administration, ROC (Taiwan): https://www.epa.gov.tw/mp.asp?mp=epaen; hospital admission data, has not deposited in publicly available repositories, are obtained from the Bureau of National Health Insurance, ROC (Taiwan). Hospital admission data is available from the corresponding author on reasonable request.

## Abbreviations

Afib: Atrial fibrillation
CAD: Coronary artery disease
Cis: Confidence intervals
CKD: Chronic kidney diseae
CO: Carbon monoxide
COPD: Chronic obstructive pulmonary disease
DM: Diabetes mellitus
HF: Heart failure
HL: Hyperlipidemia
HT: Hypertension
ICD-10: International Classification of Diseases, 10th Revision
IQR: Interquartile range
Max: Maximum
Min: Minimum
NO2: Nitrogen dioxides
ORs: Odds ratios
PM: Particle matter
PM2.5, PM10: Particulate matter ≤2.5-μm (or < 10-μm) in diameter
RH: Relative humidity
SD: Standard deviation
SO2: Sulfur dioxide
TEMP: Temperature
